# Methylation of SOCS3 in Myeloproliferative Neoplasms and Secondary Erythrocytosis/Thrombocythemia

**DOI:** 10.4274/tjh.98474

**Published:** 2013-03-05

**Authors:** Deniz Torun, Oral Nevruz, Mesut Akyol, Salih Kozan, Muhterem Bahçe, Şefik Güran, Cengiz Beyan

**Affiliations:** 1 Gülhane Military Medical Faculty, Department of Medical Genetics, Ankara, Turkey; 2 Gülhane Military Medical Faculty, Department of Hematology, Ankara, Turkey; 3 Gülhane Military Medical Faculty, Department of Biostatistics, Ankara, Turkey; 4 Gülhane Military Medical Faculty, Department of Medical Biology, Ankara, Turkey

**Keywords:** Myeloproliferative neoplasm, SOCS1, SOCS3, Secondary erythrocytosis/thrombocythemia

## Abstract

**Objective: **Myeloproliferative neoplasms (MPNs) like essential thrombocythemia (ET), polycythemia vera (PV), and primary myelofibrosis (PMF) are acquired clonal hematopoietic stem cell disorders and originate from a multipotent hematopoietic stem cell. The SOCS1 and SOCS3 genes are negative regulators of the JAK/STAT signal pathway. In this study we investigate the promoter methylation of these genes in the pathogenesis of MPNs and secondary erythrocytosis/thrombocythemia.

**Materials and Methods: **Promoter methylation of SOCS1 and SOCS3 genes was analyzed with methylation-specific PCR. PCR products were analyzed by agarose gel electrophoresis.

**Results:** No disease-specific CpG island methylation of SOCS1 was observed. Hypermethylation of the SOCS3 promoter was identified in 5 out of 19 (26.3%) PV cases, 2 out of 21 (9.5%) ET cases, 1 out of 5 (20%) PMF cases, and 9 out of 42 (21.4%) cases of secondary erythrocytosis/thrombocythemia.

**Conclusion:** The results revealed that promoter methylation of the SOCS3 gene suggests a possible role for SOCS3 methylation in the pathogenesis of MPNs and secondary erythrocytosis/thrombocythemia.

**Conflict of interest:**None declared.

## Abstract (tr)

**Amaç:** Esansiyel trombositemi (ET), polisitemia vera (PV), primer miyelofibrozis (PMF) gibi miyeloproliferatif neoplazmlar (MPN) kazanılmış klonal hematopoetik kök hücre hastalığı olup multipotent hematopoietik kök hücreden köken alırlar. SOCS1 ve SOCS3 genleri JAK/STAT sinyal yolağının negatif düzenleyicileridir. Bu çalışmada MPN ve sekonder eritrositoz/trombositemi patogenezinde bu genlerin promotor metilasyonunu incelemeyi amaçladık.

**Gereç ve Yöntemler: **SOCS1, SOCS3 genlerinin promotor metilasyonu, metilasyon spesififk PCR ile incelendi. PCR ürünleri agaroz jel elektroforezinde analiz edildi.

**Bulgular:** SOCS1 geninde CpG adacıklarında hastalıkla ilişkili metilasyon bulunamadı. 19 PV olgusunun 5’inde (%26,3), 21 ET olgusunun 2’sinde (%9,5), 5 PMF olgusunun 1’inde (%20), 42 sekonder eritrositoz/trombositemi olgusunun 9’unda (%21,4) SOCS3 promotor metilasyonu saptandı.

**Sonuç:** SOCS3 geni promotor metilasyonu MPN ve sekonder eritrositoz/trombositemi patogenezinde etkili olarak görünmektedir.

## INTRODUCTION

Myeloproliferative neoplasms (MPNs) are a group of diseases of the bone marrow in which excess cells are produced. They are related to, and may evolve into, myelodysplastic syndrome (MDS) and acute myeloid leukemia (AML), although the MPNs on the whole have a much better prognosis than these conditions. The classic MPNs are polycythemia vera (PV), essential thrombocythemia (ET), primary myelofibrosis (PMF), and chronic myelogenous leukemia (CML). They were originally grouped together based on their shared phenotype of myeloproliferation [1]. All of these neoplasms are acquired clonal hematopoietic stem cell disorders, originating from a multipotent hematopoietic stem cell. Cytogenetic and/or molecular genetic analyses are mandatory for differential diagnosis. Apart from the BCR/ABL rearrangement in CML, the JAK2 and MPL mutations play a crucial role in the pathogenesis of PV, ET, and PMF [2,3].

The JAK-STAT signaling pathway transmits information from chemical signals outside the cell, through the cell membrane, and into gene promoters on the DNA in the cell nucleus, which causes DNA transcription and activity in the cell [4]. The suppressor of cytokine signaling (SOCS) proteins inhibit the cytokine signaling cascade by using the JAK/STAT pathway in a cell [5]. Expression of SOCS1 and SOCS3 genes leads to reduced JAK and STAT phosphorylation via binding of the JH1 domain and cytokine receptor of JAK, respectively [6,7,8]. Epigenetic mechanisms such as DNA methylation regulate DNA structure and gene expression in a cell. Abnormal epigenetic mechanisms take place in the development of many diseases, including cancer. Downregulation of a gene due to methylation has been demonstrated in various studies, including the SOCS1 and SOCS3 genes [9,10,11]. 

In this study, the effects of aberrant methylation of CpG islands within the promoter region of SOCS1 and SOCS3 genes were demonstrated in the pathogenesis of PV, ET, PMF, and secondary erythrocytosis/thrombocythemia. 

## MATERIALS AND METHODS

This study was approved by the appropriate local ethics committee. Participants were ascertained according to the 2008 World Health Organization (WHO) classification system [1]. Written informed consent was obtained from all participants. A cohort of 87 patients, which included 19 cases of PV, 21 cases of ET, 5 cases of PMF, and 42 cases of secondary erythrocytosis/thrombocythemia, was enrolled to investigate the role of SOCS1 and SOCS3 promoter methylation. The control group comprised 29 healthy individuals. JAK2 V617F mutation analysis was used in differential diagnosis of MPNs and secondary erythrocytosis/thrombocythemia.

Mutation Analysis of JAK2 V617F: DNAs were isolated from peripheral blood samples in each case by using the NucleoSpin Blood Kit (Macherey-Nagel, Germany). JAK2 V617F mutation was determined in RT-PCR analyses by using a kit (JAK2 Type 1 PCR System, Dr Zeydanlı, Ankara, Turkey). The PCR conditions were 95 ^°^C for 10 min, followed by 32 cycles of 95 ^°^C for 15 s and 60 ^°^C for 1 min. 

Methylation Analysis of SOCS1 and SOCS3 CpG Islands: Genomic DNA from patients and controls were modified with sodium bisulfite using the CpGenomeTM Fast DNA Modification Kit (Chemicon International, USA and Canada). The efficacy of bisulfite modification was assessed with methylated control samples (CpG WIZ^®^ DAP-kinase Amplification Kit, Chemicon). Bisulfite-modified DNA samples were amplified by methylation-specific PCR by using methylation-specific primers and unmethylation-specific primers for the promoter region of SOCS1 and SOCS3 genes, as described by Liu et al. and Fourouclas et al., respectively [[10,11]. The primer sequences and their locations relative to the transcription start sites were noted as follows: 

SOCS1-MF 5’-TTGTTCGGAGGTCGGATTT-3’ (nt -291 to -272); SOCS1-MR 5’-ACTAAAACGCTACGAAACCG-3’ (nt -93 to -74); SOCS1-UF 5’-TTTTTTGGTGTTGTTTGGAGGTTGGATTTT-3’ (nt -301 to -272); SOCS1-UR 5’-AAAACAAAACAATAAACTA AAACACTACAAAACCA-3’ (nt -108 to -74); SOCS3-MF 5’-GAGGGGTCGTTGTTAGGAAC-3’ (nt -1265); SOCS3-MR 5’-ACAAAAACCGAAAAAAACGC-3’ (nt -1176); SOCS3-UNF 5’-GGAGGGGTTGTTGTTAGGAAT-3’ (nt -1266); SOCS3-UNR 5’-CAAAAACAAAAACCAAAAAAAACA-3’ (nt -1175) [10,11].

Bisulfite-modified DNA samples from patients and controls were amplified by PCR reaction in a total reaction volume of 25 µL containing 1X PCR buffer (Bioron, Germany), 1.5 mM MgCl2, 0.2 mM dNTP, 0.4 pmol of each primer for SOCS1, and 1 pmol of each primer for SOCS3, using 1.5 U hot-start Taq polymerase (Bioron) on a thermal cycler (Bio-Rad, USA). The PCR conditions were 95 °C for 5 min, followed by 40 cycles of 95 ^°^C for 30 s, 60 ^°^C for 40 s, and 72 ^°^C for 40 s with a final extension at 72 ^°^C for 10 min. PCR products were visualized using agarose gel electrophoresis. 

Statistical Analysis: Statistical analysis was performed using SPSS 15.00 for Windows (SPSS Inc., Chicago, IL, USA) and Microsoft Excel 2003. The Shapiro-Wilks test was used to assess normal distribution. Descriptive data are expressed as mean±standard deviation. Skewed data are shown as median and interquartile range (IQR). Chi-square (chi-square, Fisher exact, or likelihood ratio) tests were used for comparisons of JAK2 and SOCS3 among sub-groups. Categorical data are shown as numbers and percentages. The level of significance was set at p≤0.05. 

## RESULTS

JAK2 V617F mutation and methylation analysis of SOCS1/SOCS3 CpG islands were performed in 45 cases of MPNs, 42 cases of secondary erythrocytosis/thrombocythemia, and for 29 control individuals. Age, sex, and blood count characteristics of all patients and controls are summarized in Table 1. 

JAK2 V617F mutation as observed in our series is summarized in Table 2. Seventeen out of 19 PV patients (89.5%), 11 out of 21 ET patients (52.4%),and 2 out of 5 PMF patients (40.0%) revealed JAK2 V617F mutations. The JAK2 V617F mutation was not observed in secondary erythrocytosis/thrombocythemia patients or in the healthy control group (Table 2).

**Figure 1:**
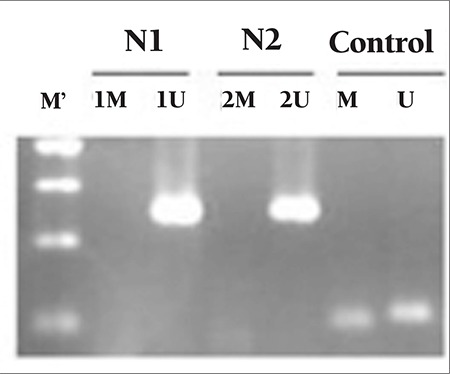
Methylation-specific PCR amplification of SOCS1 promoter region. U: unmethylated, M: methylated, M’: molecular weight marker (100 bp). N1-N2: unmethylated. Presence of bands in control groups indicates the efficacy of bisulfite treatment.

**Figure 2:**
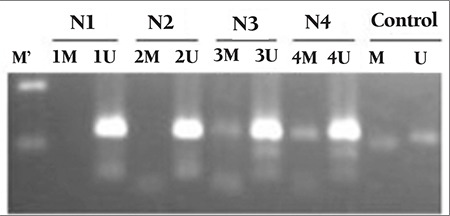
Methylation-specific PCR amplification of SOCS3 promoter region. U: unmethylated, M: methylated, M’: molecular weight marker (100 bp). N1-N2: unmethylated, N3-N4: methylated. Presence of bands in control groups indicates the efficacy of bisulfite treatment.

SOCS1 and SOCS3 CpG island methylation patterns of the study groups are presented in Table 2. The CpG islands analyzed in SOCS1 and SOCS3 genes were inside the promoter regions of these genes. MPN patients, secondary erythrocytosis/thrombocythemia patients, and the control group were negative for the methylation of the SOCS1 promoter region (Figure 1; Table 2). Five out of 19 PV patients, 2 out of 21 ET patients, 1 out of 5 ET patients, and 9 out of 42 secondary erythrocytosis/thrombocythemia patients revealed hypermethylation of the SOCS3 promoter region (Figure 2; Table 2). The control group was negative for the methylation of the SOCS3 promoter region. Four out of 5 PV patients and 2 out of 2 ET patients carrying methylation of the SOCS3 promoter region were concurrently positive for the JAK2 V617F mutation.

## DISCUSSION

Classification of MPNs was based on the clinical phenotype of these disorders and histological patterns. In recent years, many advances have occurred in the understanding and management of MPNs. Updated WHO classification of MPNs was primarily based on the proliferation capacity of the cell lines and the amount of bone marrow fibrosis. These findings were combined with clinical, laboratory, and cytogenetic/molecular genetic features [1,12].

As an acquired mutation, JAK2 V617F plays a key role in the pathogenesis of MPNs [2,13,14,15]. The JAK2 protein is a cytoplasmic tyrosine kinase and takes part in signal transduction [16]. A single amino acid substitution (valine to phenylalanine) in the JAK2 tyrosine kinase encoding gene causes uncontrolled hematopoiesis. JAK2 V617F mutation was reported in 90%-95% of PV, 50% of PMF, and 40%-50% of ET patients in the literature [17,18,19]. In our study, 89.5% of PV, 52.4% of ET, and 40.0% of PMF patients revealed JAK2 V617F mutation. These results were in line with previous reports. Secondary erythrocytosis/ thrombocythemia patients and the control group were negative for the JAK2 V617F mutation, as expected. 

Epigenetic changes, which are important for transcriptional control, may cause various diseases [20]. In our series, the SOCS1 promoter region did not reveal any methylation patterns in MPN patients, secondary erythrocytosis/thrombocythemia patients, or the control group. These findings suggest that methylation of the SOCS1 promoter region does not emerge as a molecular mechanism during the progression of MPNs and secondary erythrocytosis/thrombocythemia. These results are similar to those of a previously reported study in the literature [11]. 

SOCS3 promoter methylation represents an important mechanism in the pathogenesis of MPNs. Recently, Fourouclas et al. reported the methylation of SOCS3 in 32.0% of patients with PMF [11]. Our results show differences in some aspects. In our study, PV (26.3%) and ET (9.5%) patients also revealed SOCS3 promoter methylation, in addition to the PMF patients. This finding suggests that promoter methylation of the SOCS3 gene not only plays a role in the pathogenesis of PMF but also plays a key role in the pathogenesis of PV and ET. 

We next compared the MPNs among themselves for the frequency of SOCS3 promoter methylation. SOCS3 promoter hypermethylation did not exhibit any statistical difference among PV, ET, and PMF (p>0.05). This finding suggests that SOCS3 promoter hypermethylation may be a useful finding in the definition of MPNs but cannot be used as a tool of differential diagnosis. 

In this study, secondary erythrocytosis/thrombocythemia patients were also analyzed for SOCS3 promoter methylation. In this group, 9 out of 42 (21.4%) patients revealed aberrant SOCS3 promoter methylation. It seems that the agents that play a crucial role in the pathogenesis of secondary erythrocytosis/ thrombocythemia may show the effects through epigenetic changes. To our knowledge, the finding of SOCS3 promoter methylation in secondary erythrocytosis/ thrombocythemia has been revealed here for the first time in the literature, but long-term clinical and laboratory follow-up is needed to observe the effect of hypermethylation on the course of disease and whether or not the hypermethylation abates. 

The coincidental association of SOCS3 promoter methylation and JAK2 V617F mutation is an important finding. Four out of 5 PV patients and 2 out of 2 ET patients carrying methylation of the SOCS3 promoter region were concurrently positive for the JAK2 V617F mutation and statistical analysis revealed that the presence/absence of JAK2 V617F mutation had no effect on the existence of SOCS3 promoter methylation (p>0.05). These findings represent the fact that promoter methylation in the SOCS3 gene and the JAK2 V617F mutation might be independent. However, this finding should be supported with SOCS3 mRNA expression studies, and clinical outcomes of the patients must be compared to establish its importance in the pathogenesis and prognosis of MPN progression. 

In conclusion, SOCS3 promoter hypermethylation represents a crucial epigenetic event in the pathogenesis of MPNs and secondary erythrocytosis/thrombocythemia. For long-term clinical affects, further studies are needed. 

**Acknowledgment**

This study was supported by the Gülhane Military Medical Academy Research and Development Center, Ankara, Turkey.

## Figures and Tables

**Table 1:**
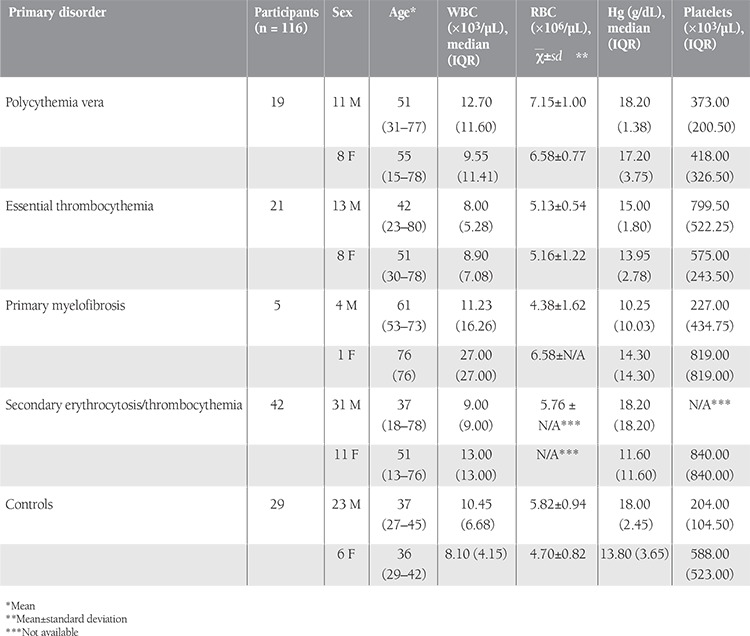
Age, sex, and blood count characteristics of the patients and control subjects.

**Table 2:**
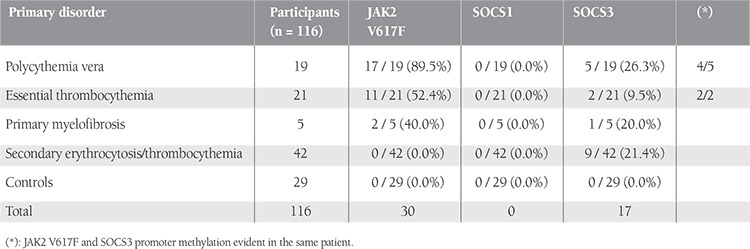
Frequency of JAK2 V617F and SOCS1/SOCS3 promoter methylation in the study groups.
